# Estradiol Reverses Ovariectomy-Induced Small RNA–mRNA Stress Signatures to Restore Neuroendocrine, Synaptic, and Immune Homeostasis in the Hypothalamus

**DOI:** 10.3390/biom16030354

**Published:** 2026-02-26

**Authors:** Muhammad Mubashir, Huan Yang, Xiaohuan Chao, Chunlei Zhang, Jiahao Chen, Yuan Ding, Hongwei Bi, Ziming Wang, Wen Guo, Junhong Fan, Mengjun Zhou, Bo Zhou

**Affiliations:** College of Animal Science and Technology, Nanjing Agricultural University, Nanjing 210095, China; 2023205057@stu.njau.edu.cn (M.M.); 2024205016@stu.njau.edu.cn (H.Y.); 2021205020@stu.njau.edu.cn (X.C.); 2020105039@stu.njau.edu.cn (C.Z.); 2023205025@stu.njau.edu.cn (J.C.); 2025205018@stu.njau.edu.cn (Y.D.); 2023805170@stu.njau.edu.cn (H.B.); 2023805171@stu.njau.edu.cn (Z.W.); 2024805170@stu.njau.edu.cn (W.G.); 2024105048@stu.njau.edu.cn (J.F.); 2024805171@stu.njau.edu.cn (M.Z.)

**Keywords:** estradiol, ovariectomy, hypothalamus, microRNA, anxiety-like behavior, neuroinflammation, gonadotropin-releasing hormone, tRF, tRNA

## Abstract

Loss of ovarian hormones following menopause or ovariectomy is associated with increased anxiety, cognitive impairment, and dysregulation of hypothalamic neuroendocrine pathways. MicroRNAs (miRNAs) and tRNA-derived fragments (tRFs) are emerging classes of small non-coding RNAs that act as post-transcriptional regulators of stress, inflammation, and synaptic function; however, their coordinated involvement in estradiol-mediated hypothalamic regulation remains poorly understood. In this study, adult female mice were assigned to control, estradiol-treated, ovariectomized (OVX), or OVX plus estradiol groups. Anxiety- and cognition-related behaviors were assessed using the open field, Y-maze, and elevated plus maze tests. Circulating estradiol levels and hypothalamic gonadotropin-releasing hormone (GnRH) expression were quantified by ELISA. Hypothalamic mRNA, miRNA, and tRF expression profiles were analyzed by RNA sequencing, followed by differential expression analysis, functional enrichment, integrative network construction, and quantitative real-time PCR validation. Ovariectomy induced anxiety-like behaviors, impaired working memory, reduced estradiol levels, and increased hypothalamic GnRH expression, all of which were reversed by estradiol treatment. Transcriptomic analysis identified 376 differentially expressed miRNAs, 182 differentially expressed tRFs, and 439 differentially expressed mRNAs, enriched in pathways related to stress responses, neuroendocrine regulation, synaptic signaling, metabolic homeostasis, and neuroinflammation. Integrated miRNA–mRNA and tRF–mRNA network analyses revealed several estradiol-responsive miRNAs (including miR-200a-5p, miR-182/183-5p, miR-381-3p, miR-148a-3p, and miR-10 family members) predicting key hub genes such as *Gcg*, *Wnt4*, *Prkacb*, *Sgk1*, *Fpr2*, and *Aldoa*, and key tRFs like tRFdb-1003, tRFdb-1013, tRFdb-1026, tRFdb-3001a and tRFdb-5020a, targeting hub genes such as *Wnt4*, *Prkacb*, *Sh3rf2*, *Hpse*, *Cxcr2* and *Zbtb16* respectively. Collectively, these findings demonstrate that estradiol ameliorates OVX-induced behavioral and endocrine dysfunction by reorganizing hypothalamic miRNA- and tRF-mediated regulatory networks involved in stress adaptation, synaptic homeostasis, and neuroimmune signaling.

## 1. Introduction

Menopause, characterized by a decline in ovarian hormone levels in humans, is associated with an increased risk of anxiety, depression, cognitive impairment, and stress-responsive neuroendocrine dysregulation [1,2]. Clinical studies indicate that reduced circulating estradiol levels alter the activity of both the hypothalamic–pituitary–adrenal (HPA) and hypothalamic–pituitary–gonadal (HPG) axes, resulting in dysfunctional glucocorticoid feedback, disrupted gonadotropin-releasing hormone (GnRH) pulsatility, and heightened inflammatory processes [3,4]. These neuroendocrine disturbances are closely linked to emotional instability and stress hypersensitivity observed during the peri- and postmenopausal periods [1,5,6].

The ovariectomy (OVX) model is widely used to mimic menopause-associated estrogen deficiency and provides a robust experimental framework for investigating the neurobiological consequences of compromised hormonal status [7]. Loss of ovarian hormones following OVX results in marked alterations in hypothalamic function, emotional behavior, cognitive performance, and metabolic homeostasis [8]. Importantly, estradiol (E2), the principal ovarian estrogen, exerts powerful neuromodulatory effects through estrogen receptors α and β, regulating gene transcription, synaptic signaling, neuropeptide release, thermoregulation, and energy balance, particularly within the hypothalamus [6]. Estradiol replacement has been shown to restore many OVX-induced behavioral and neuroendocrine abnormalities, underscoring its essential role in maintaining affective and physiological homeostasis [7,8].

At the neuroendocrine level, estradiol tightly regulates the synthesis and secretion of GnRH, the master regulator of the HPG axis. Beyond its classical reproductive functions, GnRH signaling is increasingly recognized for its involvement in stress adaptation, cognitive processes, and mood regulation [4]. Disruption of estradiol–GnRH communication following OVX may therefore contribute to both behavioral and neuroendocrine impairments associated with estrogen deficiency [9]. However, the molecular mechanisms through which estradiol restores hypothalamic stability remain incompletely understood.

MicroRNAs (miRNAs), a class of small non-coding RNAs that post-transcriptionally regulate gene expression, have emerged as critical modulators of neuronal signaling, neuroimmune balance, and adaptive stress responses [10,11]. By coordinately regulating gene networks, miRNAs influence neurotransmission, synaptic plasticity, neurotrophic signaling, and inflammatory cascades—processes central to stress resilience and emotional regulation [12]. Notably, miRNA-mediated regulatory pathways intersect with estradiol and GnRH signaling, suggesting that post-transcriptional mechanisms may play a key role in mediating estrogen-dependent hypothalamic functions [13,14]. Consistent with this concept, altered miRNA expression profiles have been reported in peri- and postmenopausal women with mood disorders, supporting a role for estrogen-sensitive miRNA–mRNA networks in emotional vulnerability and resilience [14,15].

Beyond miRNAs, tRNA-derived fragments (tRFs) have recently emerged as a biologically active class of small non-coding RNAs with regulatory functions in gene expression, stress adaptation, and cellular homeostasis [16,17]. tRFs are generated through precise cleavage of precursor or mature tRNAs and are classified into distinct subtypes based on their origin within the parent tRNA, including 5′-tRFs, 3′-tRFs, 5′- and 3′-tRNA halves (tiRNAs), and internal tRFs (i-tRFs) [18]. Accumulating evidence indicates that tRFs are not random degradation products but are selectively produced in a tissue- and condition-specific manner, particularly in response to cellular stress, inflammation, and metabolic perturbations [19,20]. Functionally, tRFs have been shown to regulate gene expression through miRNA-like mechanisms, including sequence-dependent binding to mRNA untranslated regions, modulation of translation initiation, and interaction with Argonaute proteins [21,22]. In the central nervous system, tRFs have been implicated in neuronal stress responses, synaptic plasticity, neuroinflammation, and metabolic regulation—processes that are closely linked to anxiety, depression, and cognitive dysfunction [23,24]. Notably, stress-induced tRNA halves (tiRNAs) are rapidly generated under oxidative and hormonal stress conditions and have been associated with altered neuroendocrine signaling and inflammatory pathways. Despite growing recognition of their regulatory potential, the role of tRFs in estrogen-dependent hypothalamic regulation and their contribution to ovarian hormone-related behavioral and molecular changes remain largely unexplored [25,26].

Anxiety and depression are among the most prevalent neuropsychiatric disorders worldwide, yet the molecular pathways linking hormonal status, stress regulation, and mood remain incompletely defined [27]. Dysregulation of the HPA axis can lead to aberrant glucocorticoid secretion, impaired neuronal plasticity, and emotional disturbances [28]. To establish functional relevance of molecular alterations within these systems, behavioral phenotypes must be assessed alongside endocrine and transcriptomic changes. The elevated plus maze (EPM), open field test (OFT), and Y-maze are well-established paradigms for evaluating anxiety-like behavior, exploratory activity, and cognitive flexibility, enabling direct linkage between molecular signatures and behavioral outcomes [29,30].

Despite substantial evidence supporting estradiol’s role in modulating hypothalamic circuitry and mood regulation, the post-transcriptional regulatory networks through which estradiol restores neuroendocrine and behavioral stability following estrogen loss remain poorly defined. In particular, how estradiol reshapes small RNA–mediated gene regulatory interactions to normalize stress-related, synaptic, and neuroimmune pathways in the hypothalamus is not fully understood.

Accordingly, this study tested the hypothesis that estradiol rescues ovariectomy-induced behavioral and endocrine disturbances by reorganizing hypothalamic post-transcriptional regulatory networks mediated by both microRNAs (miRNAs) and tRNA-derived fragments (tRFs). Using an integrated experimental and bioinformatic framework combining behavioral phenotyping, endocrine assessment, transcriptomic profiling, and correlation-based network analysis, we constructed estradiol-responsive miRNA–mRNA and tRF–mRNA interaction networks in the hypothalamus of control, ovariectomized, and estradiol-treated mice. This integrative analysis identified key miRNA modules—including miR-200a-5p, miR-182/183-5p, miR-148a-3p, miR-381-3p, and members of the miR-10 family—alongside several estradiol-responsive tRFs (tRFdb-1003, tRFdb-1013, tRFdb-1026, tRFdb-3001a, and tRFdb-5020a), which inversely regulate hub genes involved in stress adaptation, synaptic remodeling, metabolic signaling, and neuroimmune regulation, such as *Gcg*, *Wnt4*, *Prkacb*, *Sgk1*, *Fpr2*, *Aldoa*, *Hpse*, *Cxcr2*, and *Zbtb16*. Collectively, these findings provide mechanistic insight into estradiol-dependent small RNA–mediated regulation of hypothalamic function and highlight coordinated miRNA- and tRF-based networks as potential molecular substrates underlying menopause-associated anxiety and mood disorders.

## 2. Materials and Methods

### 2.1. Animals, Ethics Approval, and Housing Conditions

All experimental procedures were approved by the Institutional Animal Care and Use Committee (IACUC) of Nanjing Agricultural University (Nanjing, China) and were conducted in accordance with institutional and national guidelines for the care and use of laboratory animals. Every effort was made to minimize animal suffering and to reduce the number of animals used.

Sixty adult female C57BL/6J mice (8 weeks old, 20–25 g) were obtained from Jiangsu Extractive Pharmaceutical and Biotechnology Co., Ltd. (Nanjing, China). Animals were housed under controlled environmental conditions, including a 12 h light/dark cycle, a temperature of 24 ± 1 °C, and relative humidity of 44 ± 5%, with ad libitum access to food and water. Mice were allowed to acclimate to the housing environment for at least one week prior to any experimental procedures.

### 2.2. Ovariectomy and Experimental Grouping

Mice assigned to the ovariectomized (OVX) groups were anesthetized via intraperitoneal injection of tribromoethanol (0.2 mL; Nanjing AIBI Biotechnology Co., Ltd., Nanjing, China). The dorsal lumbar region was shaved and disinfected with povidone–iodine followed by 70% ethanol. Bilateral dorsal incisions were made to expose the uterine horns, after which the oviducts were ligated and the ovaries were carefully excised. The muscle layer was closed using absorbable sutures, and the skin was secured with sterile wound clips.

Postoperative care included daily monitoring for signs of distress, infection, or body weight loss exceeding 10%. After a one-week recovery period, mice were randomly assigned to one of four experimental groups (*n* = 15 per group): control (C), estradiol-treated (E2), ovariectomized (OVX), and ovariectomized plus estradiol-treated (OVX + E2).

### 2.3. Behavioral Assessments

Behavioral testing was conducted in a quiet, temperature-controlled room. To eliminate olfactory cues, all behavioral apparatuses were thoroughly cleaned with 70% ethanol between trials. Behavioral data were collected and analyzed by an experimenter blinded to group allocation.

#### 2.3.1. Elevated Plus Maze (EPM)

The elevated plus maze consisted of two open arms and two closed arms (30 × 5 cm each) elevated 50 cm above the floor. Closed arms were enclosed by opaque walls 15 cm in height. Illumination at the central platform was maintained at 50–100 lux. Each mouse was placed in the center of the maze facing an open arm and allowed to freely explore for 5–10 min. Mice that fell from the apparatus or exhibited prolonged freezing behavior (>30 s) were excluded from analysis. Time spent in the open arms and the number of open-arm entries were recorded using automated video tracking software.

#### 2.3.2. Open Field Test (OFT)

The open field apparatus consisted of a square arena (40 × 40 × 40 cm) illuminated at 100–150 lux. Mice were habituated to the testing room for 30 min prior to a 5–10 min testing session. Total distance traveled, time spent in the central zone, and thigmotaxis (preference for the peripheral area) were quantified using ANY-maze video tracking software version 7.43.

#### 2.3.3. Y-Maze Test

The Y-maze apparatus consisted of three identical arms (30 × 8 × 15 cm) positioned at 120° angles from each other. Mice were allowed to freely explore the maze for 8 min. Spontaneous alternation behavior was calculated using the formula: (number of alternations)/(total arm entries − 2) × 100, which reflects the proportion of consecutive entries into all three arms without repetition. Mice exhibiting fewer than eight total arm entries were excluded from the analysis. Behavioral testing was conducted in the following order: open field test (OFT), elevated plus maze (EPM), and Y-maze test, with a minimum interval of 24 h between consecutive tests.

### 2.4. Tissue Collection and Sample Processing

After four weeks of treatment, mice were anesthetized with tribromoethanol and euthanized by exsanguination. Blood samples were collected via cardiac puncture, allowed to clot at room temperature, and centrifuged to obtain serum, which was stored at −80 °C for subsequent hormone analyses. The hypothalamus was rapidly dissected, rinsed in ice-cold phosphate-buffered saline (PBS), snap-frozen in liquid nitrogen, and stored at −80 °C until further use. All tissues were placed into sterile 1.5 mL microcentrifuge tubes. A total of 60 biological samples (n = 15 per group) were collected for transcriptomic and miRNA analyses.

### 2.5. Estradiol Administration and Endocrine Controls

Estradiol (E2) was purchased from MedChemExpress (Nanjing, China) and dissolved in sesame oil. Mice in the OVX + E2 and E2 groups received daily intraperitoneal injections of estradiol (0.2 mg/kg body weight) for four weeks, following a previously established protocol [31]. Control and OVX mice received equivalent volumes of sesame oil as vehicle controls.

Vaginal smear cytology was performed to verify endocrine status and estrous cycle stage. Prior to behavioral testing, OVX mice were confirmed to be acyclic. Intact control and estradiol-treated mice were handled consistently to minimize variability associated with estrous cycle fluctuations.

#### 2.5.1. Serum Estradiol Measurement

Circulating estradiol concentrations were measured using a commercial enzyme-linked immunosorbent assay (ELISA) kit (Shanghai Lengton Bioscience Co., Ltd., Shanghai, China) according to the manufacturer’s instructions. The assay sensitivity was <10 pg/mL, with intra- and inter-assay coefficients of variation of <10% and <12%, respectively. Blood samples were collected between 09:00 and 11:00 to minimize diurnal variation. Serum was isolated by centrifugation at 3000× *g* for 15 min at 4 °C and stored at −80 °C until analysis.

#### 2.5.2. GnRH Quantification

GnRH levels were quantified using a validated ELISA kit (Cloud-Clone Corp., Wuhan, China) according to the manufacturer’s instructions. Mice were fasted for 5 h prior to tissue collection to reduce metabolic variability. All samples were analyzed in duplicate, and absorbance was measured at 450 nm using a microplate reader. The assay sensitivity was <5 pg/mL, with intra- and inter-assay coefficients of variation below 10%.

### 2.6. RNA Extraction and Library Preparation

Total RNA was extracted from hypothalamic tissue using TRIzol reagent (Invitrogen, Carlsbad, CA, USA) in accordance with the manufacturer’s protocol. RNA concentration and purity were assessed using a NanoDrop 2000 spectrophotometer (Thermo Scientific, Wilmington, DE, USA), and RNA integrity was evaluated with an Agilent 2100 Bioanalyzer (Agilent Technologies, Santa Clara, CA, USA). Only samples with an RNA integrity number (RIN) ≥ 7.0 were included in subsequent analyses [32].

For mRNA sequencing, ribosomal RNA was removed using the Ribo-Zero rRNA Removal Kit (Illumina, San Diego, CA, USA), and sequencing libraries were constructed using the NEBNext Ultra II RNA Library Prep Kit (New England Biolabs, South San Francisco, CA, USA) [33]. For miRNA sequencing, small RNA fragments (18–30 nt) were size-selected and ligated to 3′ and 5′ adapters using the NEBNext Small RNA Library Prep Kit. All libraries were quantified using Qubit fluorometry and quantitative PCR prior to sequencing.

### 2.7. High-Throughput Sequencing

High-throughput sequencing was performed using the Illumina NovaSeq 6000 platform. Paired-end (2 × 150 bp) sequencing was applied for mRNA libraries, and single-end (50 bp) sequencing was used for miRNA libraries. Raw sequencing reads in FASTQ format were initially assessed for quality using FastQC, followed by adapter trimming and quality filtering with Cutadapt. Only reads with a Phred quality score > 20 were retained for downstream analyses.

### 2.8. mRNA Read Alignment and Quantification

High-quality mRNA reads were aligned to the Mus musculus reference genome (GRC-M39, http://asia.ensembl.org/mus_musculus/Info/Index) accessed on 24 February using HISAT2 for spliced alignment [34,35]. Gene-level read counts were quantified using featureCounts (v2.0.6) based on Ensembl gene annotation files. To reduce background noise, only uniquely mapped reads were retained, and genes with fewer than 10 total counts across all samples were excluded from subsequent analyses.

### 2.9. MiRNA Read Processing and Quantification

miRNA sequencing data were processed using miRDeep2 [36]. Adapter sequences were removed, and the cleaned reads were aligned to the mouse reference genome as well as to mature and precursor miRNA sequences from miRBase v22.1 [37]. Raw miRNA read counts were generated using the miRDeep2 quantifier module. miRNAs with low expression levels (average counts < 5 across samples) were excluded prior to normalization and differential expression analysis.

### 2.10. Differential Expression Analysis

Differential expression analysis of mRNA and miRNA datasets was made using DESeq2 [38]. Four pairwise comparisons were conducted: control vs. ovariectomized (OVX), control vs. OVX plus estradiol (OVX + E2), estradiol-treated (E2) vs. OVX, and estradiol-treated (E2) vs. OVX + E2. Genes and miRNAs with an absolute log_2_ fold change ≥ 1 and a *p*-value ≤ 0.05 were considered significantly differentially expressed.

### 2.11. Prediction and Integration of miRNA Target Genes

Predicted target genes of differentially expressed miRNAs (DEMs) were retrieved from TargetScanMouse v9.0 (https://www.targetscan.org/mmu_80/) accessed on 30 December 2025 and miRDB v6.0 (https://mirdb.org/), while experimentally validated mRNA–miRNA interactions were obtained from miRTarBase v9.0 (https://mirtarbase.cuhk.edu.cn/~miRTarBase/miRTarBase_2025/php/index.php) accessed on 30 December 2025. To enhance biological relevance, only predicted targets that overlapped with differentially expressed genes (DEGs) identified by DESeq2 were retained for further analysis. Redundant or duplicate interactions were consolidated into unique miRNA–mRNA pairs. Venn diagram analyses were performed using VennDiagram software to identify common target genes shared among TargetScan, miRDB, and the DEG dataset, yielding a refined set of candidate miRNA–mRNA interactions associated with anxiety-, stress-, and depression-related phenotypes.

### 2.12. Identification and Differential Expression Analysis of tRNA-Derived Fragments (tRFs)

Raw small RNA sequencing data were processed to identify tRNA-derived fragments (tRFs). Adapter sequences were removed, and low-quality reads were filtered using standard small RNA-sequencing quality control pipelines. Clean reads were aligned to the mouse reference genome (mm39) and annotated tRNA sequences obtained from GtRNAdb (https://gtrnadb.org/genomes/eukaryota/Mmusc39/) accessed on 30 December 2025 using a short-read aligner, allowing no more than one mismatch. Reads mapping to annotated tRNA loci were extracted for downstream tRF analysis.

Identified tRFs were classified according to their positional origin within the parent tRNA into 5′-tRFs, 3′-tRFs, 5′-half tRFs, 3′-half tRFs, and internal tRFs (i-tRFs). tRFs with zero counts across all samples were excluded. The remaining raw counts were normalized and analyzed for differential expression using the DESeq2 package in R, which models count data based on a negative binomial distribution. tRFs with an absolute log_2_ fold change ≥ 1 and an adjusted *p*-value (Benjamini–Hochberg) ≤ 0.05 were considered significantly differentially expressed.

### 2.13. Target Prediction and tRF–mRNA Network Construction

Differentially expressed tRNA-derived fragments (tRFs) were identified using a threshold of |log_2_ fold change| ≥ 1 and adjusted *p*-value ≤ 0.05 and were selected for downstream target prediction analysis. Putative mRNA targets of significant tRFs were predicted using tRFtarget 2.0 (http://trftarget.net/) and further evaluated using RNAhybrid (https://bibiserv.cebitec.uni-bielefeld.de/rnahybrid) accessed on 30 December 2025, which assesses the thermodynamic stability of tRF–mRNA interactions. Target prediction was conducted for Mus musculus, considering potential binding sites within the 3′ untranslated region (3′-UTR), 5′ untranslated region (5′-UTR), and coding sequence (CDS) of target mRNAs. Predicted tRF–mRNA pairs were further filtered based on inverse expression patterns, retaining only negatively correlated pairs (Pearson correlation coefficient ≤ −0.50) in which upregulated tRFs corresponded to downregulated target genes and vice versa. High-confidence tRF–mRNA interactions were imported into Cytoscape (v3.9.0) for network construction and visualization. In the network, tRFs were represented as diamond-shaped nodes, whereas mRNAs were represented as elliptical nodes. Node colors indicate expression status, with red representing upregulated and green representing downregulated molecules.

### 2.14. Correlation Analysis and miRNA–mRNA Regulatory Network Construction

To infer potential regulatory relationships between DEMs and their target DEGs, normalized expression values were log_2_-transformed prior to correlation analysis. Spearman correlation coefficients (ρ) were calculated across all biological replicates for each miRNA–mRNA pair using SPSS software [39]. *p*-values were adjusted for multiple testing using the Benjamini–Hochberg false discovery rate method. MiRNA–mRNA pairs exhibiting significant negative correlations (ρ ≤ −0.5 and adjusted *p* ≤ 0.05) and inverse expression patterns (i.e., upregulated miRNA with downregulated mRNA or vice versa) were considered putative regulatory interactions and selected for network construction.

Filtered miRNA–mRNA interaction pairs were imported into Cytoscape (v3.9.0) for network visualization and topological analysis [40]. In the resulting network, miRNAs were represented as diamond-shaped nodes and mRNAs as elliptical nodes. Upregulated molecules were colored red, whereas downregulated molecules were colored green. Edge thickness was scaled according to the absolute value of the Spearman correlation coefficient, reflecting interaction strength. Hub nodes were identified based on degree centrality, highlighting miRNAs and genes with potential regulatory importance.

### 2.15. RNA Sequencing Data Validation Using Quantitative Real-Time PCR

A subset of differentially expressed miRNAs and mRNAs identified as hub nodes in the miRNA–mRNA regulatory network was validated by quantitative real-time PCR (qRT-PCR). For mRNA analysis, cDNA was synthesized using a standard reverse transcription kit (CWBIO, Nanjing, China), and gene-specific forward and reverse primers were designed using the NCBI Primer-BLAST tool (Table 1). For miRNA analysis, total RNA was polyadenylated and reverse-transcribed using the SuperStar miRNA First-Strand cDNA Synthesis Kit (poly(A) tailing method; CWBIO, Nanjing, China) according to the manufacturer’s instructions. miRNA expression was quantified using miRNA-specific forward primers and a universal reverse primer provided with the kit (Table 2). qRT-PCR was performed using SuperStar Universal SYBR Green Master Mix (CWBIO, Beijing, China) on a QuantStudio™ 10 Real-Time PCR System. Relative expression levels were calculated using the 2^−ΔΔCt^ method, with U6 small nuclear RNA and *GAPDH* used as internal reference genes for miRNA and mRNA normalization, respectively.

### 2.16. Statistical Analysis

All data are presented as mean ± standard deviation (SD) (Table S1). Statistical analyses were performed using SPSS version 25.0 (IBM, Armonk, NY, USA), and graphical representations were generated using GraphPad Prism 9 (GraphPad Software, San Diego, CA, USA). Behavioral, endocrine, and gene expression data were analyzed using one-way analysis of variance (ANOVA), followed by least significant difference (LSD) post hoc tests for multiple comparisons when appropriate. Correlations between miRNA and mRNA expression levels were evaluated using Spearman’s rank correlation analysis. For network hub validation and specific pairwise comparisons, unpaired two-tailed Student’s t-tests were applied where indicated. A *p*-value ≤ 0.05 was considered statistically significant.

## 3. Results

### 3.1. Effects of Ovariectomy and Estradiol Treatment on Hypothalamic GnRH and Circulating Estradiol Levels

**Figure 1:**
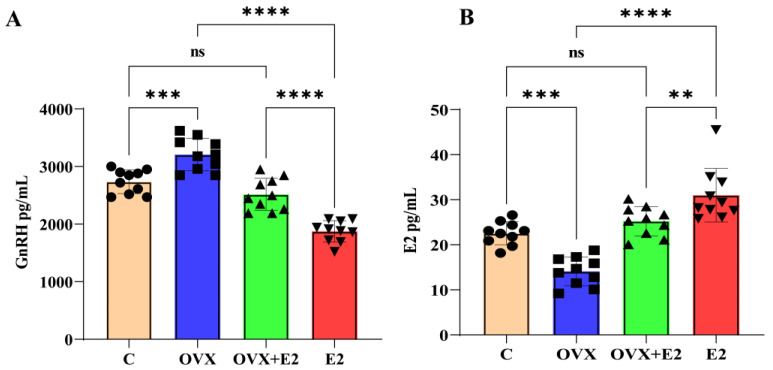
Effects of ovariectomy (OVX) and estradiol (E2) treatment on circulating estradiol and hypothalamic GnRH levels. (**A**) Hypothalamic gonadotropin-releasing hormone (GnRH) concentrations (pg/mL) in control (C), ovariectomized (OVX), ovariectomized plus estradiol-treated (OVX + E2), and estradiol-treated intact (E2) mice. OVX significantly increased hypothalamic GnRH levels compared with controls, whereas estradiol replacement markedly reduced GnRH expression. (**B**) Serum estradiol (E2) concentrations (pg/mL) in the same experimental groups. OVX significantly reduced circulating estradiol levels, while estradiol administration restored or elevated serum E2 concentrations. Data are presented as mean ± SD (n = 10 per group). Statistical significance was determined using one-way ANOVA followed by appropriate post hoc tests. ** *p* < 0.01, *** *p* < 0.001, **** *p* < 0.0001; ns, not significant.

Ovariectomy (OVX) resulted in a marked increase in hypothalamic gonadotropin-releasing hormone (GnRH) levels compared with control mice (*p* < 0.001; Figure 1A). Estradiol replacement in ovariectomized mice (OVX + E2) significantly reduced GnRH concentrations relative to the OVX group (*p* < 0.0001), restoring levels to those observed in control animals. In contrast, intact mice treated with estradiol alone (E2 group) exhibited the lowest GnRH levels among all experimental groups, which were significantly lower than those in both the control and OVX + E2 groups (*p* < 0.0001).

Circulating estradiol concentrations are shown in Figure 1B. Serum estradiol levels were significantly reduced in OVX mice compared with control animals (*p* < 0.001), confirming the effectiveness of ovariectomy. Estradiol administration significantly increased circulating estradiol levels in both the OVX + E2 and E2 groups compared with OVX mice (*p* < 0.01 and *p* < 0.0001, respectively). The intact estradiol-treated group exhibited the highest serum estradiol concentrations among all experimental groups.

### 3.2. Ovariectomy Induces Anxiety-Like Behavior in the Elevated Plus Maze and Is Reversed by Estradiol

**Figure 2:**
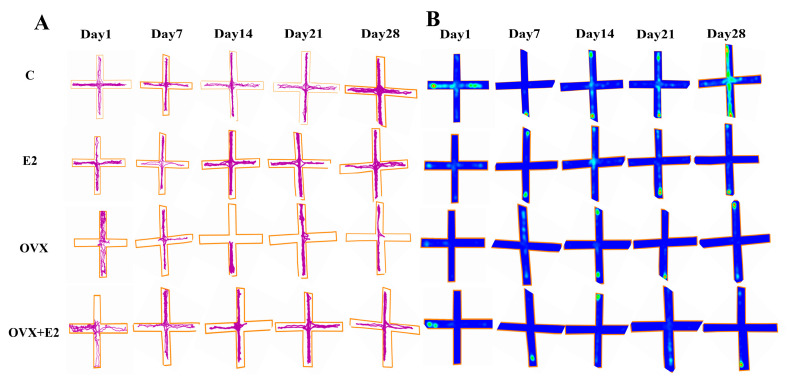
Open-field test trajectories and spatial occupancy heat maps across experimental groups. (**A**) Representative locomotor trajectories illustrating exploratory behavior of mice in the open-field arena on Days 1, 7, 14, 21, and 28 in the control (C), estradiol-treated (E2), ovariectomized (OVX), and ovariectomized plus estradiol-treated (OVX + E2) groups. Movement paths reflect overall locomotor activity and exploratory behavior during the testing period. (**B**) Corresponding spatial occupancy heat maps depicting the distribution of time spent within the open-field arena. Warmer colors (red–yellow) indicate increased occupancy, whereas cooler colors (blue–green) represent reduced exploration. The central zone is outlined to facilitate assessment of anxiety-like behavior. Behavioral tracking and heat-map generation were performed using ANY-maze software (Stoelting Co., Wood Dale, IL, USA).

In the elevated plus maze (EPM), ovariectomized (OVX) mice exhibited pronounced anxiety-like behavior, as evidenced by a significant reduction in both open-arm time and the number of open-arm entries compared with control mice (*p* < 0.05). Estradiol treatment effectively reversed these behavioral deficits: both OVX + E2 and estradiol-treated intact (E2) mice showed significantly increased open-arm exploration relative to OVX mice (*p* < 0.05), with values approaching those observed in the control group. These findings indicate that estradiol deficiency promotes anxiety-like behavior, whereas estradiol replacement restores exploratory behavior and reduces anxiety. Representative movement trajectories of individual mice and the corresponding spatial occupancy heat maps illustrating exploratory patterns are shown in Figure 2A,B, respectively.

### 3.3. Ovariectomy Reduces Center Exploration in the Open Field Test Without Affecting Locomotion

**Figure 3:**
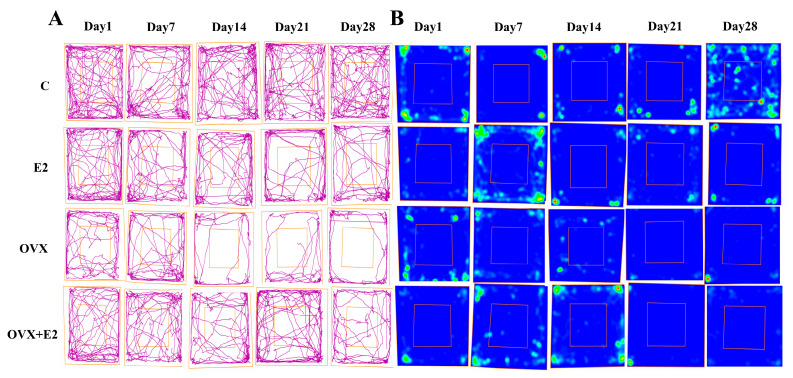
Open-field test trajectories and spatial occupancy heat maps across experimental groups. (**A**) Representative locomotor trajectories illustrating exploratory behavior of mice in the open-field arena on Days 1, 7, 14, 21, and 28 in the control (C), estradiol-treated (E2), ovariectomized (OVX), and ovariectomized plus estradiol-treated (OVX + E2) groups. Movement paths reflect overall locomotor activity and exploratory behavior during the test session. (**B**) Corresponding spatial occupancy heat maps depicting the distribution of time spent within the open-field arena. Warmer colors (red–yellow) indicate increased occupancy, whereas cooler colors (blue–green) represent reduced exploration. The central zone is outlined to facilitate assessment of anxiety-like behavior. Behavioral tracking and heat-map generation were performed using ANY-maze software (Stoelting Co., Wood Dale, IL, USA).

During the OFT, OVX mice spent significantly less time in the center zone and exhibited increased thigmotaxis compared with control mice (*p* < 0.05), indicative of heightened anxiety-like behavior. Importantly, total distance traveled did not differ among groups, demonstrating that locomotor activity was not affected and did not confound the anxiety-related measures. Estradiol treatment significantly increased center exploration and reduced thigmotaxis in OVX mice (*p* < 0.05), consistent with an anxiolytic-like effect. Representative locomotor trajectories of individual mice are shown in Figure 3A, and the corresponding spatial occupancy heat maps illustrating exploratory patterns are presented in Figure 3B.

### 3.4. Estradiol Repairs Working Memory Impairment Caused by OVX in the Y-Maze

**Figure 4:**
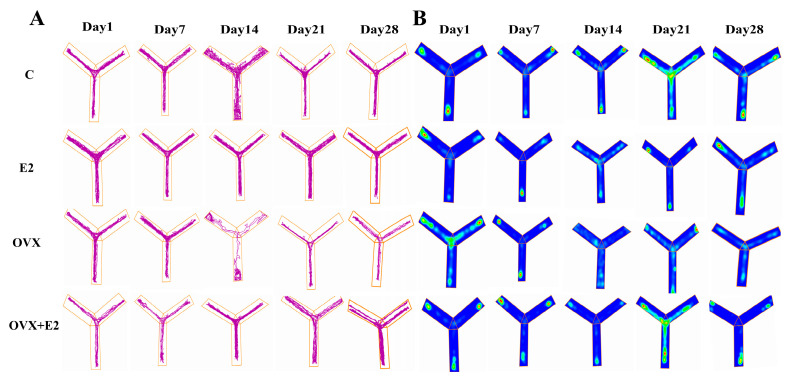
Y-maze test assessing spatial working memory across experimental groups. (**A**) Representative movement trajectories of mice in the Y-maze on Days 1, 7, 14, 21, and 28 for the control (C), estradiol-treated (E2), ovariectomized (OVX), and ovariectomized plus estradiol-treated (OVX + E2) groups. Distinct trajectory shapes correspond to individual arm entries and exploratory movement patterns recorded during the test session. (**B**) Corresponding occupancy heat maps showing the spatial distribution of time spent in each arm of the Y-maze, with warmer colors (red–yellow) indicating greater occupancy and cooler colors (blue–green) indicating reduced exploration. Behavioral recording, trajectory tracking, and heat-map generation were performed using automated video-tracking software (ANY-maze, Stoelting Co., Wood Dale, IL, USA). Spontaneous alternation behavior was used as an index of spatial working memory.

Performance in the Y-maze revealed a significant reduction in spontaneous alternation percentage in OVX mice compared with control animals (*p* < 0.05), indicating impaired spatial working memory. Estradiol replacement significantly improved alternation behavior in OVX + E2 mice relative to OVX animals (*p* < 0.05), restoring performance toward control levels. Representative movement trajectories of individual mice are shown in Figure 4A, and corresponding occupancy heat maps illustrating arm exploration patterns are presented in Figure 4B.

### 3.5. Ovariectomy Alters Hypothalamic Gene Expression Profiles Involved in Neuroendocrine, Immune, and Metabolic Regulation

**Figure 5:**
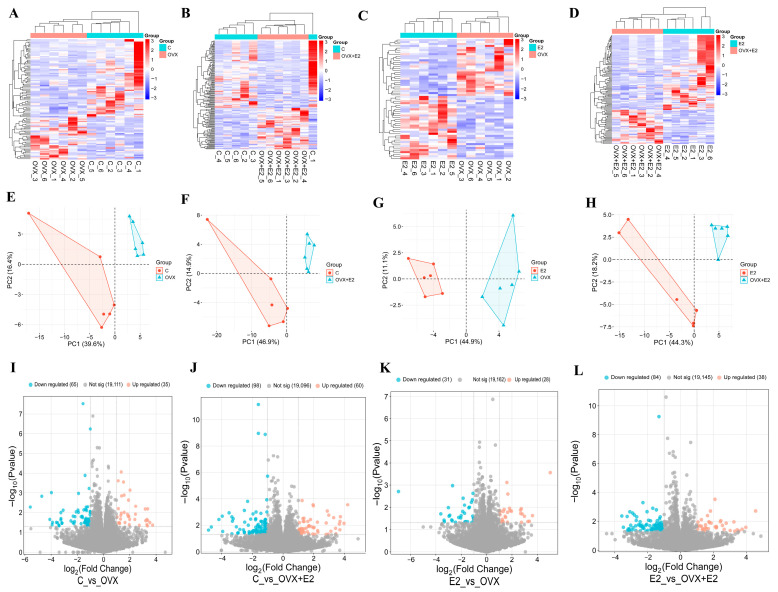
Transcriptomic profiling, PCA clustering, and differential expression analysis of hypothalamic mRNAs. (**A**–**D**) Heatmaps showing hierarchical clustering of significantly altered mRNAs for each pairwise comparison: (**A**) control (C) vs. OVX, (**B**) C vs. OVX + E2, (**C**) E2 vs. OVX, and (**D**) E2 vs. OVX + E2. Columns represent individual samples and rows represent genes. Red and blue indicate higher and lower expression levels, respectively. (**E**–**H**) Principal component analysis (PCA) plots illustrating sample separation based on global mRNA expression profiles for the corresponding comparisons. Distinct clustering indicates transcriptional reprogramming induced by ovariectomy and modulation by estradiol treatment. (**I**–**L**) Volcano plots displaying the distribution of differentially expressed mRNAs for each comparison. The *x*-axis represents log_2_ fold change and the *y*-axis indicates −log_10_ (*p*-value). Upregulated genes are shown in red, downregulated genes in blue, and non-significant genes in gray.

Transcriptome sequencing identified a total of 439 differentially expressed genes (DEGs) across all group comparisons (|log_2_FC| ≥ 1, *p* < 0.05), including 278 downregulated and 161 upregulated genes. Ovariectomy induced pronounced transcriptional alterations characterized by downregulation of neuroendocrine and stress-adaptive genes, including *Gcg*, *Sgk1*, *Fpr2*, *Bdnf*, and *Wnt3*, alongside upregulation of metabolic and inflammatory genes such as *Aldoa*, *Wnt4*, and *Btn2a2*. Estradiol replacement (OVX + E2) reversed many OVX-induced transcriptional changes, restoring the expression of neuroendocrine- and immune-related genes while further enhancing the expression of *Aldoa*, *Wnt4*, and *Sh3rf2*, which are associated with synaptic remodeling and metabolic support. Principal component analysis (PCA) and hierarchical clustering demonstrated clear separation among control, OVX, E2, and OVX + E2 groups, confirming distinct transcriptional states associated with ovarian hormone status (Figure 5). Hierarchical clustering revealed increased sample consistency following normalization, and boxplot analysis confirmed uniform signal distributions across samples (Supplementary Figure S1A–H).

### 3.6. Differentially Expressed miRNAs Define an Estradiol-Sensitive Post-Transcriptional Signature

**Figure 6:**
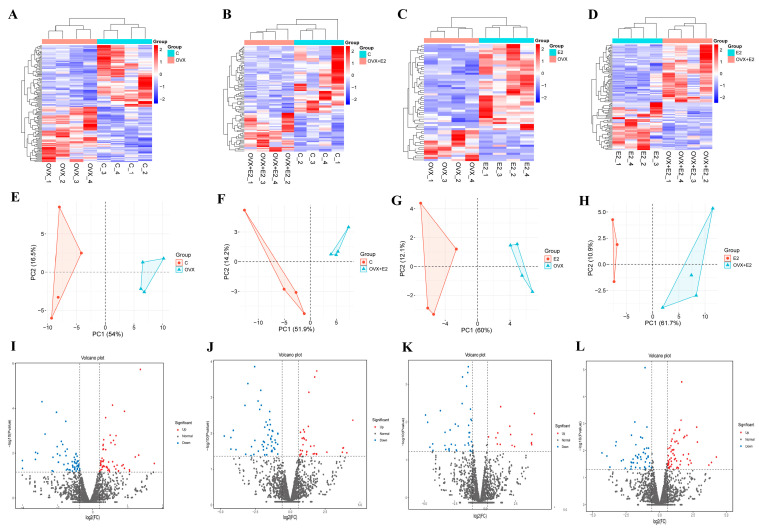
miRNA expression profiling, PCA clustering, and differential expression analysis across experimental groups. (**A**–**D**) Heatmaps showing hierarchical clustering of differentially expressed miRNAs for the pairwise comparisons: (**A**) control (C) vs. OVX, (**B**) C vs. OVX + E2, (**C**) E2 vs. OVX, and (**D**) E2 vs. OVX + E2. Columns represent individual samples and rows represent miRNAs. Red and blue indicate higher and lower expression levels, respectively. (**E**–**H**) Principal component analysis (PCA) plots illustrating separation of experimental groups based on global miRNA expression profiles. Distinct clustering patterns demonstrate transcriptional divergence following ovariectomy and partial restoration toward control-like profiles after estradiol supplementation. (**I**–**L**) Volcano plots showing the relationship between log_2_ fold change (*x*-axis) and −log_10_ (*p*-value) (*y*-axis) for each pairwise comparison. Upregulated and downregulated miRNAs are shown in red and blue, respectively, whereas non-significant changes are shown in gray.

A total of 376 differentially expressed miRNAs (DEMs) were identified across all group comparisons, including 167 upregulated and 209 downregulated miRNAs. Ovariectomy (OVX) induced a stress-associated miRNA expression profile characterized by increased levels of miR-200a-5p, miR-148a-3p, miR-206-3p, miR-192-3p, and miR-381-3p, alongside downregulation of the synaptic plasticity–associated miR-182-5p. Estradiol replacement normalized the hypothalamic miRNA landscape, restoring the expression of miR-182-5p, miR-183-5p, and members of the miR-10 family, while suppressing miRNAs elevated by OVX. Principal component analysis (PCA) and hierarchical clustering revealed marked separation between OVX and estradiol-treated groups, indicating estrogen-dependent modulation of hypothalamic miRNA expression patterns (Figure 6). Following normalization, miRNA transcriptome probe signals were uniformly distributed, as demonstrated by boxplot analysis, and hierarchical clustering showed increased sample consistency (Supplementary Figure S2A–H).

### 3.7. Global Expression Profiling of Differentially Expressed tRNA-Derived Fragments

**Figure 7:**
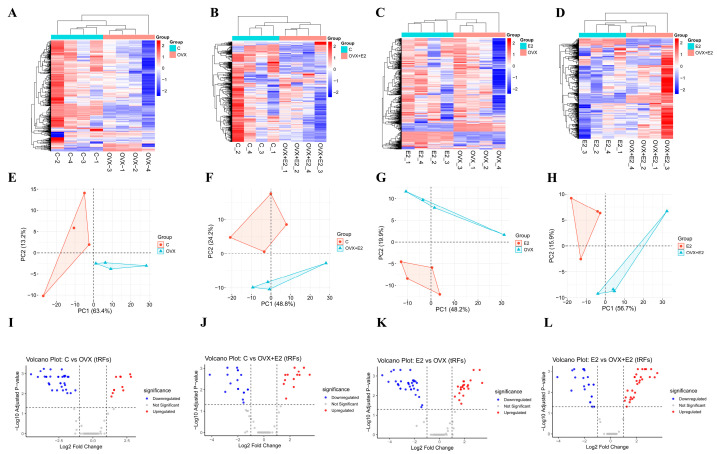
Expression profiling of tRNA-derived fragments (tRFs) in the hypothalamus across experimental groups. (**A**–**D**) Heatmaps showing normalized expression (z-score) of differentially expressed tRFs (DE-tRFs) in comparisons between (**A**) Control (C) vs. Ovariectomized (OVX), (**B**) C vs. OVX + E2, (**C**) E2 vs. OVX, and (**D**) E2 vs. OVX + E2 groups. Rows represent individual tRFs; columns represent biological replicates. Color scale: Red = upregulated, Blue = downregulated, White = unchanged. (**E**–**H**) Principal Component Analysis (PCA) plots of tRF expression profiles, showing separation between groups based on the top 2 principal components (PC1 and PC2). (**E**) C vs. OVX, (**F**) C vs. OVX + E2, (**G**) E2 vs. OVX, (**H**) E2 vs. OVX + E2. Ellipses indicate 95% confidence intervals. (**I**–**L**) Volcano plots showing DE-tRFs in each comparison. *x*-axis: Log2 (Fold Change); *y*-axis: −Log10 (Adjusted *p*-value). Red dots: Significantly upregulated tRFs (Adjusted *p* < 0.05, |Log2(FC)| > 1); Blue dots: Significantly downregulated tRFs; Gray dots: Non-significant tRFs.

Using a threshold of |log_2_ fold change| ≥ 1 and adjusted *p*-value ≤ 0.05, 182 tRNA-derived fragments (tRFs) were identified as differentially expressed, comprising 75 upregulated and 107 downregulated tRFs across experimental groups. Hierarchical clustering heatmaps (Figure 7A–D) showed clear and distinct tRF expression patterns among the Control (C), OVX, E2, and OVX + E2 groups, with strong intra-group clustering and marked inter-group separation. Ovariectomy induced substantial alterations in tRF expression, while estradiol treatment partially restored these changes, particularly in the OVX + E2 group. Principal component analysis (PCA; Figure 7E–H) demonstrated clear segregation of OVX samples from controls, with E2-treated groups shifting toward the control cluster, indicating estrogen-dependent modulation of global tRF expression profiles. Volcano plots (Figure 7I–L) revealed significant up- and down-regulation of multiple tRFs across pairwise comparisons, highlighting pronounced transcriptional changes following ovariectomy and their modulation by estradiol treatment. Sample clustering and dendrogram analyses (Supplementary Figure S3A–H). further confirmed the robustness and consistency of tRF expression patterns across biological replicates.

### 3.8. Integration of miRNA and mRNA Profiles Identifies Predicted Regulatory Pairs

**Figure 8:**
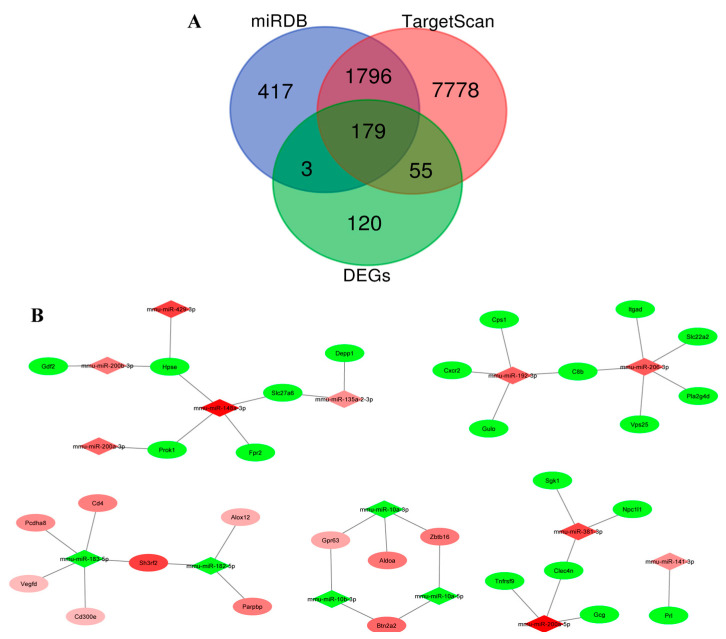
Prediction and visualization of the hypothalamic miRNA–mRNA regulatory network following estradiol replacement. (**A**) Venn diagram illustrating the overlap between differentially expressed genes and predicted miRNA targets identified by TargetScan and miRDB. (**B**) Network visualization depicting differentially expressed miRNAs (diamond-shaped nodes) and their mRNA targets (elliptical nodes) in control, OVX, and estradiol-treated mice. Red and green nodes indicate upregulated and downregulated molecules, respectively.

Integration of DEMs with DEGs using TargetScan, miRDB, miRTarBase, and correlation analysis identified 41 predicted miRNA–mRNA regulatory pairs that met the criteria of inverse expression, dual-database support, and significant negative correlation (ρ ≤ −0.5, *p* < 0.05). These interactions included OVX-associated inflammatory and stress-related regulatory axes, such as miR-200a-5p–*Gcg*/*Tnfrsf9*, miR-148a-3p–*Fpr2*/*Hpse*, miR-381-3p–*Sgk1*, and miR-206-3p–*Bdnf*/*Wnt3*, as well as estradiol-responsive synaptic and metabolic interactions, including miR-182/183-5p–*Wnt4*/*Prkacb*/*Sh3rf2* and miR-10a/10b–*Aldoa*/*Btn2a2*. These regulatory pairs formed the core of the hypothalamic miRNA–mRNA interaction network underlying OVX-induced dysfunction and estradiol-mediated restoration (Figure 8A,B).

### 3.9. Functional Enrichment Analysis Reveals Coordinated Regulation of Stress, Immune, and Synaptic Pathways

**Figure 9:**
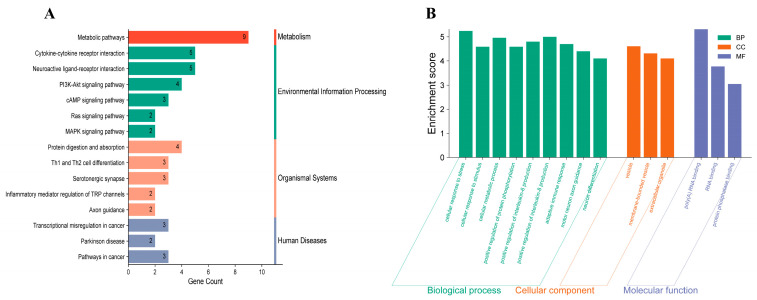
Functional enrichment analysis of miRNA target genes. (**A**) Top 15 significantly enriched KEGG pathways (*p* < 0.05) for predicted miRNA target genes, highlighting neuroendocrine, synaptic, and immune regulatory pathways. (**B**) Top 15 significantly enriched Gene Ontology (GO) terms (*p* < 0.05), emphasizing processes including apoptosis regulation, cytokine production, astrocyte activation, and synaptic signaling.

Functional enrichment analysis of the 41-gene regulatory set using Gene Ontology (GO) and Kyoto Encyclopedia of Genes and Genomes (KEGG) pathway analysis revealed significant enrichment in pathways associated with stress responses, neuroendocrine signaling, immune regulation, and synaptic function. Enriched KEGG pathways included neuroactive ligand–receptor interaction, serotonergic synapse signaling, MAPK, PI3K–Akt, and cAMP signaling pathways, as well as inflammatory mediator-regulated transient receptor potential (TRP) channel modulation. GO enrichment analysis highlighted biological processes related to negative regulation of apoptosis, interleukin-6 and interleukin-8 production, astrocyte activation, axon guidance, and dopaminergic synaptic transmission. Together, these findings indicate coordinated, estradiol-sensitive post-transcriptional regulation of neuroimmune homeostasis and neuronal plasticity in the hypothalamus (Figure 9).

### 3.10. miRNA–mRNA Regulatory Network Distinguishes OVX-Associated Pathological Modules from Estradiol-Restored Protective Modules

A targeted miRNA–mRNA regulatory network was constructed using the top differentially expressed miRNAs and validated target genes, revealing two distinct functional modules. The OVX-associated pathological module was dominated by upregulated miRNAs, including miR-200a-5p, miR-148a-3p, miR-200b-3p, miR-206-3p, and miR-192-3p, which targeted genes such as *Clec4n*, *Hpse*, *Fpr2*, *Slc27a6*, *Prok1*, *Gdf2*, *Wnt3*, and *Cxcr2*. These interactions were primarily linked to inflammatory signaling, extracellular matrix remodeling, complement activation, chemokine signaling, and disrupted neuroendocrine communication.

In contrast, the estradiol-restored protective module consisted of miRNAs normalized by E2 treatment, notably miR-182/183-5p and members of the miR-10a/10b family, which targeted genes including *Sh3rf2*, *Wnt4*, *Prkacb*, *Aldoa*, *Btn2a2*, *Zbtb16*, *Gpr63*, and *Vegfd*. These regulatory interactions were enriched in pathways related to synaptic organization, metabolic support, transcriptional regulation, and immune resolution, reflecting coordinated molecular restoration following estradiol replacement.

### 3.11. tRF–mRNA Regulatory Network Analysis

The resulting network revealed that several tRFs potentially regulate key genes involved in neuroplasticity, inflammation, and stress signaling. Notably, tRFdb-1001 showed predicted interactions with Bdnf and *Cxcr2*, suggesting a potential role in modulating neurotrophic support and chemokine-mediated inflammatory signaling. tRFdb-1026 was linked to Wnt3, *Hpse*, and *Cxcr2*, indicating possible involvement in Wnt signaling and extracellular matrix remodeling, both of which are implicated in stress-induced neural and immune responses. Additionally, tRFdb-3001a and tRFdb-3004b were associated with genes such as Prok1, Gulo, and Depp1, which are related to metabolic regulation and cellular stress responses.

In the downregulated gene module, tRFdb-1003, tRFdb-1013, tRFdb-5002a and tRFdb-5020a were predicted to target genes including *Prkacb*, *Wnt4*, *Vegfd*, *Btn2a2*, *Zbtb16*, *Sh3rf2*, and *Gpr63*. These genes play important roles in intracellular signaling, angiogenesis, immune modulation, and neuronal development, processes that are frequently disrupted under chronic stress and depressive conditions.

**Figure 10:**
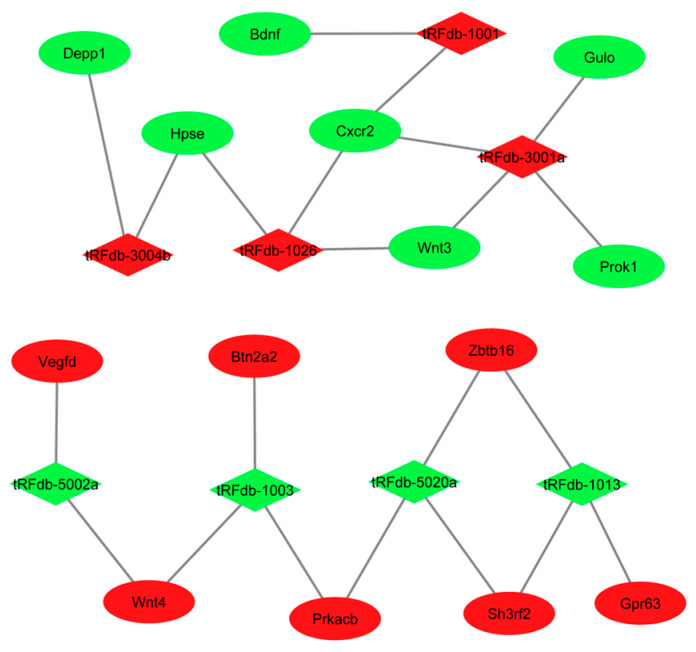
Predicted tRF-mRNA regulatory network. Network of differentially expressed tRFs (red diamonds) and their mRNA targets (green circles: upregulated; blue circles: downregulated) in control, OVX, and estradiol-treated mice. Nodes are sized by interaction degree; edges represent predicted targeting (seed complementarity, MFE ≤ −20 kcal/mol).

Overall, the tRF–mRNA interaction network highlights a subset of differentially expressed tRFs that may act as post-transcriptional regulators of genes relevant to stress response, neuroinflammation, and depression-related molecular pathways. These findings support the emerging concept that tRFs function in a miRNA-like manner and may contribute to the fine-tuning of gene expression in stress-associated neurobiological processes (Figure 10).

### 3.12. GO and KEGG Enrichment Analysis of tRNA-Derived Fragments (tRFs)

**Figure 11:**
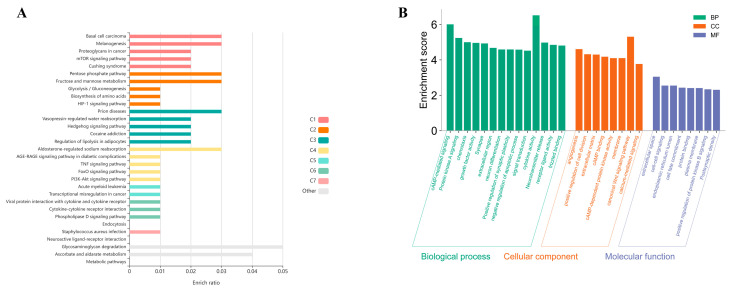
Functional enrichment analysis of tRF target genes. (**A**) KEGG pathway enrichment dot plot. Pathways are colored by functional cluster (C1–C7) and sized by enrichment ratio (dot diameter); the *x*-axis indicates enrichment ratio (gene count/background gene count). Top pathways include the pentose phosphate pathway, HIF-1 signaling, and mTOR signaling. (**B**) GO enrichment bar plot. Terms are grouped by category: biological process (BP, green), cellular component (CC, orange), and molecular function (MF, blue). The *y*-axis shows enrichment score (−log_10_ (adjusted *p*-value)), with top terms including axon guidance (BP), synapse (CC), and protein kinase A binding (MF). All enrichments were calculated using KOBAS (GO and KEGG) with adjusted *p* < 0.05.

GO and KEGG enrichment analyses were conducted to elucidate the functional roles of predicted target genes of differentially expressed tRFs (Figure 11). KEGG analysis showed significant enrichment in pathways related to neuroendocrine signaling, inflammation, and metabolic regulation, including neuroactive ligand–receptor interaction, TNF signaling, PI3K–Akt, FoxO, and HIF-1 signaling pathways. Metabolism-associated pathways such as glycolysis/gluconeogenesis, pentose phosphate pathway, and hormone-regulated ion and water reabsorption were also enriched, indicating a link between tRFs and stress-associated metabolic adaptation.

GO analysis further revealed enrichment of target genes involved in cell–cell signaling, synaptic signaling, neuronal differentiation, and regulation of apoptotic processes (Biological Process). Enriched Cellular Component terms were mainly associated with the plasma membrane and extracellular region, while Molecular Function terms included receptor binding, protein kinase activity, and calcium ion binding. Collectively, these findings suggest that tRFs may regulate key molecular pathways underlying neuroinflammation, synaptic plasticity, and stress-related behavioral responses in this study (Figure 11).

### 3.13. qRT-PCR Validation and Correlation Analysis of OVX and Estradiol-Regulated miRNA–mRNA Pairs

**Figure 12:**
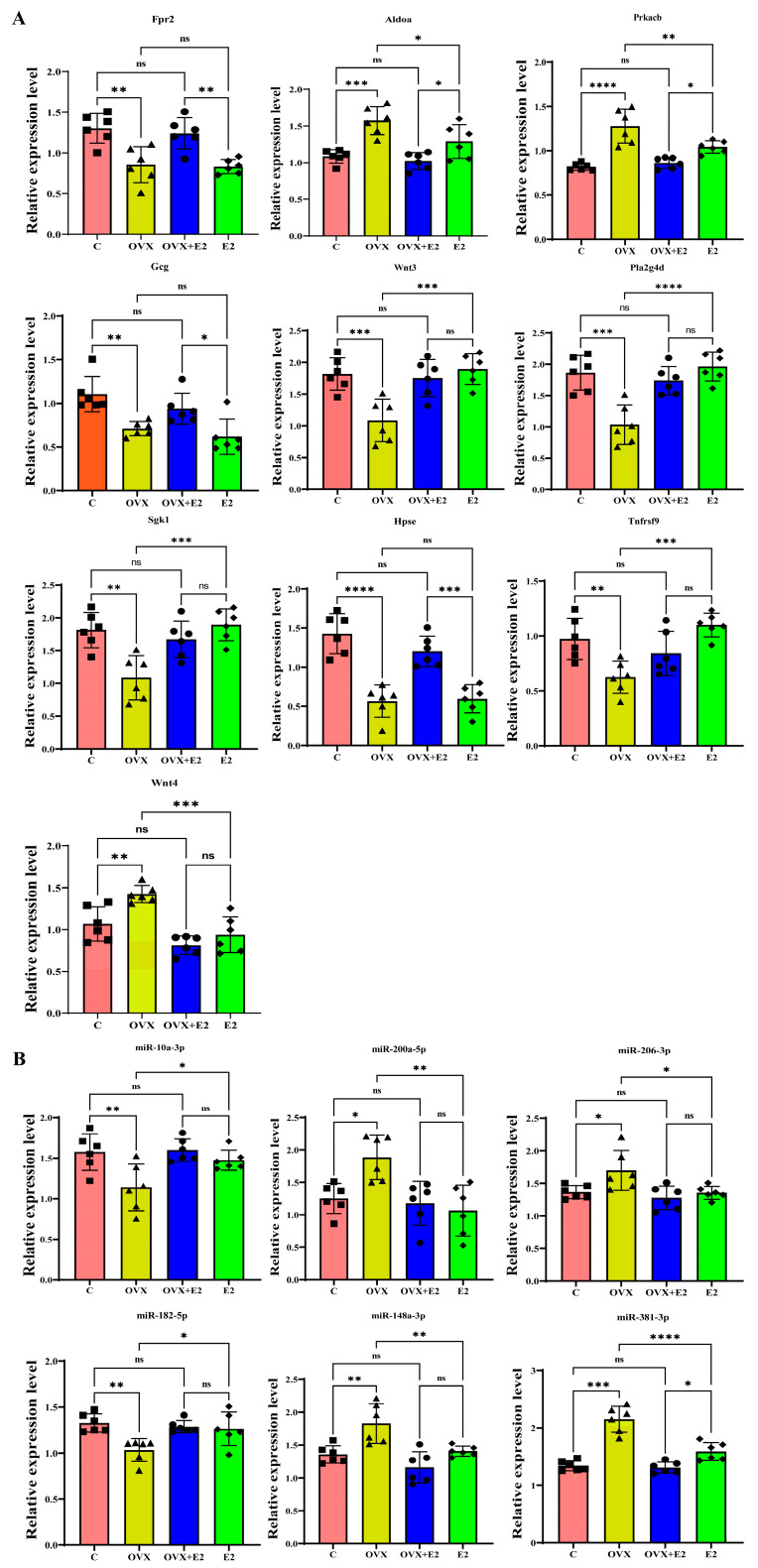
qRT-PCR validation of hypothalamic mRNA and miRNA expression. Relative expression levels of target genes and miRNAs were quantified by qRT-PCR and normalized to endogenous controls (GAPDH for mRNAs, U6 for miRNAs) across control (C), OVX, OVX + E2, and E2 groups. (**A**) Expression of validated mRNAs. OVX significantly downregulated *Fpr2*, *Gcg*, *Wnt3*, *Pla2g4d*, *Sgk1*, *Hpse*, and *Tnfrsf9*, while *Aldoa*, *Prkacb*, and *Wnt4* were significantly upregulated compared with controls. Estradiol treatment restored these expression levels toward baseline. (**B**) Expression of validated miRNAs. OVX significantly upregulated miR-200a-5p, miR-206-3p, miR-148a-3p, and miR-381-3p, whereas miR-10a-3p and miR-182-5p were significantly downregulated relative to controls. Estradiol replacement normalized miRNA expression. Data are presented as mean ± SD (n = 6 per group). Statistical significance was determined using one-way ANOVA followed by appropriate post hoc tests. * *p* < 0.05, ** *p* < 0.01, *** *p* < 0.001, **** *p* < 0.0001; ns, not significant.

qRT-PCR confirmed OVX-induced downregulation of *Fpr2*, *Gcg*, *Wnt3*, *Pla2g4d*, *Sgk1*, *Hpse*, and *Tnfrsf9*, alongside upregulation of *Aldoa*, *Prkacb*, and *Wnt4*, consistent with transcriptomic findings. Estradiol treatment significantly reversed these changes. miRNA qRT-PCR further demonstrated OVX-induced upregulation of miR-200a-5p, miR-148a-3p, miR-206-3p, and miR-381-3p, together with downregulation of miR-182-5p and miR-10a-3p, all of which were normalized by estradiol therapy (Figure 12).

**Figure 13:**
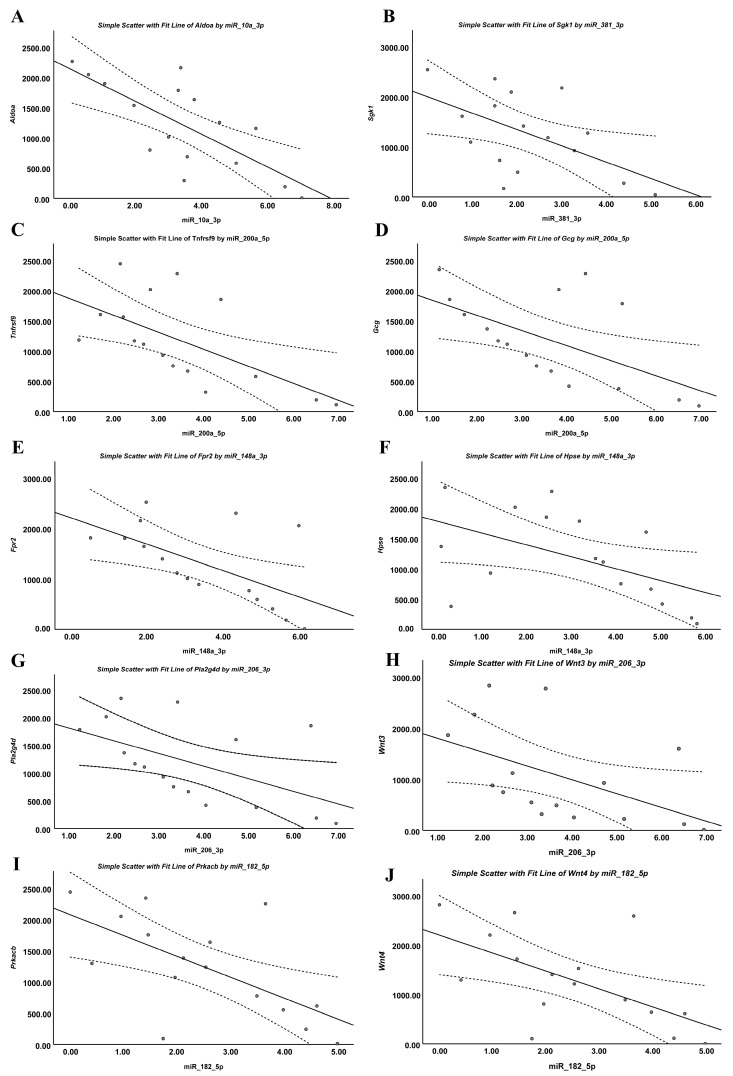
Spearman correlation analysis of validated miRNA–mRNA pairs. Scatter plots illustrate significant negative correlations between selected miRNAs and the mRNA levels of their predicted target genes. (**A**–**J**) miR-10a-3p, miR-381-3p, miR-200a-5p, miR-148a-3p, miR-206-3p, and miR-182-5p were inversely correlated with *Aldoa*, *Sgk1*, *Tnfrsf9*, *Gcg*, *Fpr2*, *Hpse*, *Pla2g4d*, *Wnt3*, *Prkacb*, and *Wnt4*, respectively (*p* < 0.05), validating the predicted miRNA–mRNA regulatory interactions underlying OVX-induced hypothalamic dysregulation and estradiol-mediated molecular restoration. The solid line represents the fitted regression line, and the dotted lines denote the 95% confidence interval of the Spearman correlation.

Spearman correlation analysis revealed strong inverse relationships between predicted miRNA–mRNA pairs, validating the regulatory interactions: miR-10a-3p–*Aldoa*, miR-381-3p–*Sgk1*, miR-200a-5p–*Gcg* and *Tnfrsf9*, miR-148a-3p–*Hpse* and *Fpr2*, miR-206-3p–*Pla2g4d* and *Wnt3*, miR-182-5p–*Prkacb* and *Wnt4*. All correlations were significant (*p* < 0.05), supporting the predicted miRNA–mRNA regulatory network underlying OVX-induced hypothalamic dysfunction and estradiol-mediated molecular restoration (Figure 13).

## 4. Discussion

Anxiety, chronic stress, and depressive symptoms remain major contributors to the global neuropsychiatric burden, profoundly affecting emotional regulation, cognition, and overall quality of life. At the molecular level, these behavioral disturbances result from maladaptive interactions between neuroendocrine, synaptic, and inflammatory systems, with the hypothalamus serving as a central hub integrating stress reactivity and mood regulation. Dysregulation of the hypothalamic–pituitary–adrenal (HPA) axis is a hallmark of stress-related disorders, and mounting evidence implicates altered glucocorticoid signaling, impaired synaptic plasticity, and neuroimmune activation in the pathogenesis of anxiety- and depression-like behaviors. miRNAs act as critical post-transcriptional regulators that fine-tune gene expression and play essential roles in neuronal excitability, neurotrophic signaling, and inflammatory homeostasis. Despite their importance, the miRNA–mRNA networks linking estrogen deficiency to affective dysfunction remain largely unexplored. Ovariectomy (OVX), a well-established model of estrogen withdrawal, mimics human menopausal vulnerability by inducing anxiety-like behavior, metabolic disturbances, and heightened neuroinflammation. However, the post-transcriptional mechanisms connecting estrogen loss to disrupted hypothalamic homeostasis are not fully understood.

In this study, we combined transcriptomic profiling with comprehensive behavioral phenotyping to identify estrogen-sensitive miRNA–mRNA regulatory modules and to elucidate how E2 restores molecular and emotional stability. Our findings reveal that OVX triggers distinct pathological miRNA–mRNA signatures associated with stress, neuroimmune activation, and synaptic dysfunction, while estradiol replacement reorganizes these networks, normalizing gene expression, synaptic signaling, and behavioral outcomes. In addition to miRNAs, this study identifies tRNA-derived fragments (tRFs), also referred to under standardized nomenclature as tRNA-derived RNAs (tDRs), as estrogen-sensitive post-transcriptional regulators in the hypothalamus. tRFs are generated through precise cleavage of mature or precursor tRNAs and are now recognized as functional small RNAs rather than random degradation products. Increasing evidence demonstrates that tRFs participate in translational control, mRNA destabilization, stress signaling, and immune regulation, particularly in contexts of hormonal fluctuation and cellular stress.

Among the OVX-induced miRNA changes, miR-200a-5p and miR-148a-3p emerged as major pathological regulators, exhibiting strong upregulation and clear inverse relationships with several stress-adaptive genes, including *Gcg*, *Fpr2*, and *Sgk1* [41,42]. Consistent with prior studies implicating the miR-200 family in glucocorticoid dysregulation and heightened anxiety-like behavior, our findings indicate that elevated miR-200a-5p selectively suppresses genes critical for hypothalamic feedback control of the HPA axis [43]. *Gcg*, which encodes neuropeptides involved in metabolic signaling and stress adaptation, was significantly reduced in OVX mice, reflecting diminished neuroendocrine resilience [44]. Similarly, *Fpr2*, a receptor with anti-inflammatory and neuroprotective properties, was downregulated, consistent with previous reports linking reduced *Fpr2* signaling to neuroinflammation and anxiety [45,46]. Downregulation of *Sgk1*, a glucocorticoid-responsive kinase essential for neural stress adaptation, further supports the notion that miR-200a-5p overexpression impairs stress-buffering processes [47,48]. At the behavioral level, suppression of these targets was associated with increased thigmotaxis, reduced open-arm exploration in the EPM, and impaired cognitive flexibility in the Y-maze. OVX mice consistently avoided the center zone in the OFT and open arms in the EPM, reflecting heightened threat sensitivity and overactivation of stress-related circuits [49]. These behavioral phenotypes coincided with OVX-induced suppression of key neuroendocrine regulators (*Gcg*, *Sgk1*) and downregulation of neuroimmune genes (*Fpr2*, *Tnfrsf9*, *Clec4n*), supporting the link between miRNA dysregulation, neuroinflammation, and emotional vulnerability [50,51].

Importantly, estradiol replacement restored behavioral performance across all paradigms, reactivating stress-adaptive pathways, enhancing synaptic remodeling, and attenuating inflammatory signaling [52,53]. The concurrent normalization of miRNA expression, particularly miR-182/183-5p and miR-10 family members, supports a model in which estradiol reconfigures hypothalamic circuits via coordinated post-transcriptional regulation. These findings highlight behavioral outcomes as functional readouts of miRNA–mRNA network states, demonstrating that hormonal status directly shapes emotional and cognitive behavior through integrated molecular programs.

Another miRNA significantly affected by OVX was miR-148a-3p, which showed marked upregulation and negative associations with genes involved in neuroimmune regulation, including *Fpr2*, *Prok1*, and *Hpse*. Previous studies have identified miR-148a as a critical regulator of immunological signaling, capable of modulating microglial activation and altering neuroimmune tone under stress [54]. In our study, elevated miR-148a-3p corresponded with decreased expression of *Prok1* and *Hpse*, genes involved in immune resolution and antigen processing [55]. Suppression of *Fpr2* by miR-148a-3p indicates a convergent mechanism by which multiple miRNAs enhance neuroinflammatory signaling in the absence of estrogen [56]. Estradiol treatment reversed these changes, restoring *Fpr2*, *Prok1*, and *Hpse* expression in parallel with improvements in anxiety-like and exploratory behaviors. Collectively, these results reinforce the concept that estrogen maintains emotional stability, in part, by restraining inflammation-associated miRNAs and promoting neuroimmune homeostasis.

In the OVX group, we observed robust upregulation of multiple miRNAs, including miR-200a-5p, miR-148a-3p, miR-206-3p, miR-192-3p, miR-381-3p, miR-429-3p, miR-141, and miR-135a-2-3p, forming a stress- and inflammation-associated signature. Notably, miR-148a has been implicated in panic disorder and anxiety-related pathways in humans, while the miR-200 family is altered in chronic stress and depression models and is upregulated in learned helplessness rats [57,58,59]. Similarly, miR-206-3p is elevated in depression and chronic stress paradigms and contributes to anxiety- and depression-like behaviors via suppression of *Bdnf*, Wnt signaling, and other plasticity-related targets [60,61,62]. Together, these findings indicate that OVX drives a hypothalamic miRNA profile consistent with heightened stress susceptibility, neuroinflammatory bias, and affective dysregulation. Other members of this cluster, including miR-381, miR-429, miR-135a-2-3p, and miR-141, are less directly characterized in mood regulation but possess predicted targets in neuroplasticity and immune pathways, suggesting additional contributions to OVX-induced vulnerability.

Conversely, estradiol treatment restored or increased the levels of miR-182-5p, miR-183-5p, and miR-10 family members, which in our network were linked to synaptic, metabolic, and immune-regulatory genes such as *Wnt4*, *Prkacb*, *Sh3rf2*, *Aldoa*, *Btn2a2*, *Gpr63*, and *Zbtb16*. Notably, downregulation of miR-182-5p in OVX mice may permit the disinhibition of targets such as *Wnt4* and *Prkacb*, thereby enhancing Wnt–cAMP/PKA signaling critical for synaptic homeostasis and emotional regulation under estradiol modulation. miR-182-5p has been variably implicated in stress and depression models, with knockdown sometimes alleviating depression-like behavior [63]. In our paradigm, estradiol restored OVX-suppressed miR-182-5p concomitant with improved anxiety-like and cognitive behaviors, suggesting that precise tuning of this miRNA, rather than absolute up- or downregulation, is crucial for hypothalamic resilience.

Although less extensively characterized in affective disorders, miR-183 and miR-10 family members are recognized regulators of neuronal plasticity, differentiation, and immune-metabolic signaling, supporting the interpretation that estradiol reorganizes post-transcriptional networks to promote synaptic stability and neuroendocrine recovery [12,64,65]. Restoration of the miR-183/96/182 cluster following estradiol treatment suggests reactivation of pathways required for neuronal remodeling and structural resilience. Prior studies have shown that increased expression of this cluster enhances long-term memory and object recognition in rodents, whereas knockdown impairs memory performance [66,67]. Moreover, activity-dependent downregulation of miR-182 in the amygdala facilitates synaptic actin remodeling and fear-memory consolidation, highlighting its role in structural plasticity [67]. In our data, recovery of miR-182/183 coincided with normalization of key targets such as *Wnt4*, *Sh3rf2*, and *Parpbp*, supporting renewed synaptic signaling and metabolic support for neuronal function. Although miR-182/183 are not universally defined as “antidepressant miRNAs,” their estradiol-mediated restoration, alongside improved cognitive and exploratory behaviors, aligns with prior evidence on cluster-mediated plasticity and suggests that balanced expression of this module underlies estrogen-dependent neural resilience.

Estradiol treatment also restored expression of the miR-10 family (miR-10a/b-5p, miR-10a/b-3p), which had been downregulated following OVX. Members of the miR-10 family are evolutionarily conserved and classically recognized for regulating Hox gene clusters, developmental patterning, and differentiation programs [68,69]. Emerging evidence suggests that miR-10 contributes to neural differentiation by modulating pathways such as Wnt signaling and focal adhesion, which are critical for neuronal maturation and may influence neuron–glia interactions [70,71]. In our study, estradiol-mediated restoration of miR-10 coincided with normalization of potential targets, including *Btn2a2*, *Gpr63*, and *Zbtb16*, genes implicated in immunological co-stimulation, neuronal signaling, and transcriptional regulation, respectively. These findings indicate that miR-10 family members may facilitate metabolic balance, transcriptional flexibility, and coordinated neuroimmune modulation under estrogen control. However, given the limited direct evidence linking miR-10 to mood or stress-related outcomes, this interpretation should be considered a mechanistic hypothesis that warrants further experimental validation.

Although the primary aim of this study was to assess estrogen deficiency and replacement after ovariectomy, inclusion of an estradiol-treated intact (E2) group was critical for contextualizing estrogen’s central effects within a physiologically intact hypothalamic-pituitary-gonadal (HPG) axis. Estradiol administration to intact mice produced the expected negative feedback suppression of hypothalamic GnRH expression, confirming functional estrogen responsiveness of the neuroendocrine circuitry. Importantly, comparison of the E2-only and OVX + E2 groups revealed context-dependent effects of estradiol. In healthy mice, estradiol primarily exerted homeostatic feedback on GnRH, whereas in OVX + E2 mice, estradiol restored molecular and behavioral deficits caused by estrogen deprivation. This distinction is critical for interpreting OVX-related alterations as pathological consequences of hormone loss rather than generic effects of estradiol, thereby strengthening causal inferences regarding therapeutic restoration.

Integrative analysis of transcriptomic profiles, miRNA expression patterns, and regulatory network architecture indicates that estradiol maintains hypothalamic homeostasis at a systems level rather than through isolated pathways. Estrogen withdrawal disrupted multiple physiological domains, including neuroendocrine signaling (*Gcg*, *Sgk1*), neuroimmune control (*Fpr2*, *Tnfrsf9*), synaptic remodeling (*Wnt4*, *Sh3rf2*, *Bdnf*), and metabolic resilience (*Aldoa*). These domains were mediated by distinct but interrelated miRNA modules: OVX-upregulated miR-200a-5p and miR-148a-3p enhanced inflammatory and stress-related signaling, whereas estradiol-restored miR-182/183-5p and miR-10 family members supported synaptic integrity and metabolic recovery. The concurrent normalization of molecular signatures and behavioral outcomes following estradiol treatment suggests that E2 restores coordinated communication between the HPA and HPG axes, suppresses pro-inflammatory signaling, and reinstates synaptic plasticity. These results are consistent with prior reports of estrogen-dependent regulation of neuronal excitability, microglial phenotype, mitochondrial function, and glucocorticoid sensitivity. Importantly, our study expands this framework by identifying specific miRNA–mRNA interaction pairs that mediate the transition from an OVX-induced stress-vulnerable state to an estradiol-restored resilient phenotype. Collectively, these findings support a mechanistic paradigm in which estrogen deprivation induces anxiety-like behavior, cognitive deficits, and neuroendocrine instability through coordinated dysregulation of hypothalamic miRNA–mRNA networks, while estradiol re-establishes molecular and behavioral homeostasis through network-level post-transcriptional regulation.

Estradiol restores emotional and cognitive stability by re-establishing disrupted biochemical modules. Several of the miRNAs and genes identified in this study—including miR-200a-5p, miR-182-5p, and miR-10 family targets such as *Clec4n*, *Wnt4*, *Btn2a2*, and *Zbtb16*—have also been reported to change in menopausal women, underscoring the translational relevance of these findings. The network-based methodology applied here provides a powerful strategy for identifying biomarkers associated with estrogen withdrawal-related mood vulnerability and highlights prospective therapeutic targets for hormone-responsive anxiety, stress, depression, and cognitive impairment. By integrating miRNA and mRNA data, our approach extends beyond traditional transcriptome analysis, offering mechanistic insight into how estradiol modulates stress resilience at the post-transcriptional level.

The tRFs incorporated into the present regulatory network—tRFdb-1003 (tDR-T1:T31-Ser-GCT-4-1), tRFdb-1013 (tDR-T1:T28-Pro-CGG-1-3), tRFdb-1001 (tDR-T1:T20-Ser-TGA-1-1), tRFdb-1026 (tDR-T1:T30-Cys-GCA-3-1), tRFdb-3001a (tDR-61:76-Asn-GTT-2-M2), tRFdb-3004b (tDR-55:76-Cys-GCA-2-M4), tRFdb-5002a (tDR-1:23-Gly-GCC-1), and tRFdb-5020a (tDR-1:19-Glu-TTC-1)—represent distinct fragment classes derived from specific cleavage positions within their parental tRNAs. The use of both tRFdb identifiers and standardized tDR nomenclature ensures consistency with earlier studies while aligning with current classification systems, thereby strengthening biological interpretation and reproducibility.

Previous studies have demonstrated that tRNA-derived fragments originating from specific parental tRNAs are preferentially induced under conditions of cellular stress, inflammation, and hormonal perturbation, suggesting conserved functional roles rather than stochastic degradation [72]. In particular, tRFs derived from tRNA-Ser, tRNA-Gly, tRNA-Cys, and tRNA-Glu—parental sources represented among the estradiol-responsive fragments identified in the present study—have been repeatedly implicated in stress-adaptive and immune-regulatory processes. Stress-induced cleavage of these tRNAs has been reported in neural, immune, and endocrine tissues, where the resulting tRFs modulate translational repression, mRNA stability, and inflammatory signaling cascades [16]. tRFs derived from tRNA-Ser, including tRFdb-1003 (tDR-T1:T31-Ser-GCT-4-1) and tRFdb-1001 (tDR-T1:T20-Ser-TGA-1-1), have been associated with stress-responsive translational control and neuronal signaling plasticity. Serine tRNA-derived fragments have been shown to accumulate in response to oxidative stress and glucocorticoid exposure, conditions that are closely linked to hypothalamic–pituitary–adrenal (HPA) axis dysregulation and anxiety-like behaviors [22]. The involvement of these fragments in regulating targets such as *Wnt4* and *Prkacb* in the present study aligns with prior evidence connecting serine-associated tRFs to pathways governing synaptic remodeling, intracellular signaling, and hormone-dependent neuronal adaptation.

Similarly, glycine- and cysteine-derived tRFs, including tRFdb-5002a (tDR-1:23-Gly-GCC-1), tRFdb-1026 (tDR-T1:T30-Cys-GCA-3-1), and tRFdb-3004b (tDR-55:76-Cys-GCA-2-M4), have been reported to participate in immune modulation and redox-sensitive signaling. Glycine tRNA-derived fragments have been linked to inflammatory regulation and metabolic homeostasis, while cysteine-derived tRFs are frequently enriched under conditions of oxidative stress and neuroinflammation [73]. In the context of the present study, the predicted targeting of *Hpse* and *Cxcr2* by these fragments provides a mechanistic link between estrogen deficiency, extracellular matrix remodeling, chemokine signaling, and neuroimmune activation—hallmarks of stress-related affective disorders.

tRFdb-5020a (tDR-1:19-Glu-TTC-1), derived from tRNA-Glu, further reinforces this framework, as glutamate-related tRFs have been implicated in neuronal excitability, synaptic transmission, and inflammatory signaling in previous reports. Dysregulation of glutamatergic pathways is a well-established feature of anxiety and depression, and the estradiol-sensitive expression of Glu-derived tRFs observed here suggests a potential role in modulating excitatory–inhibitory balance within the hypothalamus [74]. Likewise, the Asn-derived fragment tRFdb-3001a (tDR-61:76-Asn-GTT-2-M2) may contribute to translational control under stress conditions, consistent with reports linking Asn tRNA cleavage to adaptive responses during cellular stress and metabolic challenge.

Functionally, several of these tRFs have been implicated in gene regulatory processes analogous to miRNA-mediated repression, including sequence-specific interactions with mRNA 3′ untranslated regions. In the present study, target prediction and inverse correlation analysis revealed that estradiol-responsive tRFs converge on hub genes involved in neuroendocrine signaling, synaptic plasticity, metabolic regulation, and neuroimmune activation [75,76]. Notably, tRFs targeting *Wnt4* and *Prkacb* suggest a role in estrogen-sensitive Wnt signaling and cAMP-dependent protein kinase pathways, both of which are essential for synaptic remodeling and stress adaptation. Similarly, tRF-mediated regulation of *Hpse* and *Cxcr2* links these fragments to extracellular matrix remodeling and chemokine signaling, processes increasingly recognized in neuroinflammation and mood disorder pathophysiology. Targeting of *Zbtb16* and *Sh3rf2* further implicates tRFs in transcriptional regulation and neuronal signaling stability.

Importantly, the differential expression of these tRFs following ovariectomy and their normalization upon estradiol replacement suggest that tRF biogenesis and function are hormonally regulated in the hypothalamus. This observation is consistent with emerging literature showing that tRF expression is dynamically modulated by endocrine cues and stress conditions, positioning tRFs as adaptive regulators rather than static byproducts of tRNA metabolism. The coordinated regulation of overlapping target genes by both miRNAs and tRFs observed in this study supports a model in which estradiol orchestrates a multilayered small RNA regulatory network to maintain hypothalamic homeostasis.

Despite providing integrated behavioral, endocrine, and transcriptomic evidence that estradiol reorganizes hypothalamic post-transcriptional regulatory networks disrupted by ovariectomy, several limitations should be acknowledged. First, the identified miRNA–mRNA and tRF–mRNA interactions were inferred from bioinformatic target prediction, inverse correlation analysis, and limited qRT-PCR validation; direct functional validation using miRNA/tRF mimics, inhibitors, or luciferase reporter assays was not performed. Therefore, these interactions should be interpreted as predictive regulatory associations rather than definitive causal mechanisms. Second, while tRNA-derived fragments were identified as estradiol-responsive regulators, the precise biogenesis pathways, subcellular localization, and molecular mechanisms through which individual tRFs influence mRNA stability or translation were not experimentally examined and remain to be clarified in future studies. Third, immune and inflammatory alterations were primarily inferred from transcriptomic enrichment analyses, without complementary protein-level or histological validation of glial activation (e.g., astrocytic or microglial markers), which would further substantiate neuroimmune involvement. Fourth, although anxiety- and cognition-related behaviors were assessed using the open field, elevated plus maze, and Y-maze tests, direct correlations between individual behavioral measures and molecular expression profiles were not modeled. Finally, estradiol was administered via daily intraperitoneal injection at a dose that may produce supraphysiological hormone fluctuations and may not fully recapitulate physiological estrogen replacement; alternative delivery approaches, such as sustained-release implants, could provide more stable endocrine conditions in future investigations. Addressing these limitations will be essential for strengthening mechanistic insight into estradiol-dependent small RNA regulation of stress-related hypothalamic circuits.

## 5. Conclusions

Ovarian hormone deficiency disrupts hypothalamic neuroendocrine, metabolic, and immune homeostasis, leading to anxiety- and stress-related behavioral impairments, whereas estradiol replacement restores these functions. Ovariectomy reduced circulating estradiol levels, increased hypothalamic GnRH expression, and altered stress- and immune-related gene expression. Integrated transcriptomic analyses revealed estradiol-sensitive miRNA– and tRF–mRNA regulatory networks, involving miR-182-5p, miR-381-3p, miR-206-3p, while key tRFdb-1003, tRFdb-1013, tRFdb-3001a and tRFdb-5020a targeting *Wnt4*, *Prkacb*, *Sgk1*, *Bdnf*, and *Cxcr2*. These coordinated small RNA networks represent promising molecular targets for estrogen-related affective and cognitive disorders.

## Figures and Tables

**Table 1:** Primers for real-time quantitative PCR.

Gene	Gene ID	Primer Sequence (5′-3′)	Product Length/bp
*GAPDH*	NM_001289726.2	F: ACCCTTAAGAGGGATGCTGC	130
		R: CCCAATACGGCCAAATCCGT	
*Gcg*	NM_008100.4	F: CACCAAGAGGAACCGGAACA	265
		R: GATGAAGTCCCTGGTGGCAA	
*Tnfrsf9*	NM_011612.2	F: GTTCCAGCTGCCACTATTCTTCTTC	750
		R: CCAGTGGTCTTCTTAAATGGTTGC	
*Wnt3*	NM_009521.3	F: GCAAGTAGTGAGCCAGGGCA	362
		R: TAGCCCAGCCTGTTCTGTTG	
*Fpr2*	NM_008039.2	F: AGACCTCAGCTGGTTGTGCAG	405
		R: CACAAATGCAGCGGTCCAAG	
*Aldoa*	NM_001177307.2	F: CGCGTTCGCTCCTTAGTCCT	236
		R: GTGGCAGTGCTTTCCTTTCCTA	
*Prkacb*	NM_011100.5	F: GGACGGGTTCCTTTGGAAGA R: GTGATGGGAACCGGACCTTT	630
*Pla2g4d*	NM_001024137.1	F: CACCCGAGAGACTACATGGC R: CCCACAACAGGGACCTCATC	958
*Hpse*	NM_152803.5	F: GAACCCCAGACTTACGGTGG R: AAACTGTTGGGCTCATTGCC	109
*Wnt4*	NM_009523.2	F: CGAGCAATTGGCTGTACCTG R: GGGAGTCCAGTGTGGAACAG	219
*Sgk1*	NM_001161845.2	F: CAAAAACAGCTCTCCTCCAGC	526
		R: TGATCCATCTTCGTACCCGTT	

**Table 2:** Forward primer for real-time quantitative PCR.

MicroRNA	Primer Sequence (5′-3′)
mmu-miR-200a-5p	CATCTTACCGGACAGTGCTGGA
mmu-miR-206-3p	TGGAATGTAAGGAAGTGTGTGG
mmu-miR-148a-3p	TCAGTGCACTACAGAACTTTGT
mmu-miR-182-5p	TTTGGCAATGGTAGAACTCACA
mmu-miR-381-3p	TATACAAGGGAAGCTTTCTTGT
mmu-miR-10a-3p	CAAATTCGTATCTAGGGGAATA
U6	TGGCCCCTGCGCAAGGATG

## Data Availability

The original contributions presented in this study are included in the article/Supplementary Materials. Further inquiries can be directed to the corresponding author.

## Supplementary Materials

The following supporting information can be downloaded at: https://www.mdpi.com/article/10.3390/biom16030354/s1, Figure S1: Quality control, normalization, and sample clustering across experimental groups; Figure S2: Quality control and clustering analysis of hypothalamic miRNA sequencing data; Figure S3: Quality control, normalization, and clustering analysis of hypothalamic tRNA-derived fragment (tRF) sequencing data; Table S1: Relative expression level of mRNA and miRNA detected in stress, anxiety and depression; Table S2: Sample allocation table.

## Abbreviations

The following abbreviations are used in this manuscript:

ANOVA	Analysis Of Variance
DEGs	Differentially Expressed Genes
EPM	Elevated Plus Maze
ELISA	Enzyme-Linked Immunosorbent Assay
E2	Estradiol
FASTQ	Format for High-Throughput Sequencing Data
GO	Gene Ontology
GnRH	Gonadotropin-Releasing Hormone
HPA Axis	Hypothalamic–Pituitary–Adrenal Axis
HPG Axis	Hypothalamic–Pituitary–Gonadal Axis
KEGG	Kyoto Encyclopedia of Gene and Genome
OVX	Ovariectomy
OFT	Open Field Test
tRFs	Transfer RNA-related Fragments
