# Supplementary material for: Estradiol Reverses Ovariectomy-Induced Small RNA–mRNA Stress Signatures to Restore Neuroendocrine, Synaptic, and Immune Homeostasis in the Hypothalamus
Source: Biomolecules. 2026 Feb 26;16(3):354. doi: 10.3390/biom16030354 (PMC13023533; doi:10.3390/biom16030354)
Supplement: Supplementary file 1 [file biomolecules-16-00354-s001.zip › Table S1, S2.pdf]

Table S1. Relative expression level of mRNA and miRNA detected in Stress, Anxiety and Depression

**mRNA/miRNA**	**Control (C)**	**E2**	**OVX**	**OVX+E2**	**P-Value**	**Regulation trend**
mmu-miR-200a-5p	1.22±0.23	1.06±0.39	1.88±0.34	1.17±0.33	<0.001	Up
mmu-miR-206-3p	1.37±0.09	1.28±0.22	1.69±0.30	1.27±0.20	<0.002	Up
mmu-miR-148a-3p	1.35±0.12	1.40±0.07	1.82±0.30	1.16±0.23	<0.0002	Up
mmu-miR-182-5p	1.37±0.09	1.28±0.22	0.59±0.34	1.17±0.31	<0.005	Down
mmu-miR-381-3p	1.34±0.08	1.59±0.15	2.14±0.22	1.31±0.09	<0.0001	Up
mmu-miR-10a-3p	1.57±0.22	1.47±0.12	1.13±0.30	1.59±0.13	<0.002	Down
Fpr2	1.30± 0.18	0.83±0.087	0.85±0.22	1.24±0.19	<0.0001	Down
Gcg	1.10±0.20	0.61±0.20	0.70±0.08	0.93±0.17	<0.0004	Down
Tnfrsf9	0.97±0.18	1.1±0.10	0.62±0.14	0.84±0.19	<0.0005	Down
Aldoa	1.08±0.09	1.24±0.15	1.57±0.19	1.02±0.11	<0.0001	Up
Sgk1	1.81±0.26	1.89±0.24	1.08±0.33	1.67±0.28	<0.0003	Down
Pla2g4d	1.86±0.27	1.96±0.23	1.03±0.31	1.73±0.22	<0.0001	Down
Hpse	1.42±0.25	0.59±0.17	0.56±0.20	1.20±0.19	<0.0001	Down
Prkacb	0.81±0.03	1.27±0.19	0.86±0.06	1.04±0.06	<0.0001	Up
Wnt3	1.81±0.25	1.08±0.33	1.75±0.29	1.89±0.24	<0.0003	Down
Wnt4	1.06±0.20	1.42±0.10	0.81±0.11	0.93±0.21	<0.0001	Up

Table S2. Sample Allocation Table

Experimental Analysis	Animals per Group
Behavioral tests	n = 15
Hormone assays (E2, GnRH)	n = 10
mRNA-seq	n = 6
miRNA-seq	n = 4
RT-qPCR	n = 6
Correlation	n = 16
